# Episodic Memory Encoding and Retrieval in Face-Name Paired Paradigm: An *f*NIRS Study

**DOI:** 10.3390/brainsci11070951

**Published:** 2021-07-19

**Authors:** Qian Yu, Boris Cheval, Benjamin Becker, Fabian Herold, Chetwyn C. H. Chan, Yvonne N. Delevoye-Turrell, Ségolène M. R. Guérin, Paul Loprinzi, Notger Mueller, Liye Zou

**Affiliations:** 1 Exercise Psychophysiology Laboratory, Institute of KEEP Collaborative Innovation, School of Psychology, Shenzhen University, Shenzhen 518060, China; yuqianmiss@163.com; 2 Swiss Center for Affective Sciences, University of Geneva, 1205 Geneva, Switzerland; Boris.Cheval@unige.ch; 3 Laboratory for the Study of Emotion Elicitation and Expression (E3Lab), Department of Psychology, FPSE, University of Geneva, 1205 Geneva, Switzerland; 4 MOE Key Laboratory for Neuroinformation, The Clinical Hospital of Chengdu Brain Science Institute, School of Life Science and Technology, University of Electronic Science and Technology of China, Xiyuan Ave 2006, Chengdu 611731, China; ben_becker@gmx.de; 5 Department of Neurology, Medical Faculty, Otto von Guericke University, Leipziger Street 44, 39120 Magdeburg, Germany; fabian.herold@st.ovgu.de (F.H.); notger.mueller@dzne.de (N.M.); 6 German Center for Neurodegenerative Diseases (DZNE), Research Group Neuroprotection, Leipziger Street 44, 39120 Magdeburg, Germany; 7 Department of Psychology, The Education University of Hong Kong, Tai Po, Hong Kong, China; cchchan@eduhk.hk; 8 UMR 9193-SCALab-Sciences Cognitives et Sciences Affectives, Université de Lille, F-59000 Lille, France; yvonne.delevoye@univ-lille.fr (Y.N.D.-T.); segolene.guerin@univ-lille.fr (S.M.R.G.); 9 Department of Health, Exercise Science, and Recreation Management, The University of Mississippi, Oxford, MS 38677, USA; pdloprin@olemiss.edu

**Keywords:** episodic memory, face-name paired paradigm, *f*NIRS

## Abstract

Background: Episodic memory (EM) is particularly sensitive to pathological conditions and aging. In a neurocognitive context, the paired-associate learning (PAL) paradigm, which requires participants to learn and recall associations between stimuli, has been used to measure EM. The present study aimed to explore whether functional near-infrared spectroscopy (*f*NIRS) can be employed to determine cortical activity underlying encoding and retrieval. Moreover, we examined whether and how different aspects of task (i.e., novelty, difficulty) affects those cortical activities. Methods: Twenty-two male college students (age: M = 20.55, SD = 1.62) underwent a face-name PAL paradigm under 40-channel *f*NIRS covering fronto-parietal and middle occipital regions. Results: A decreased activity during encoding in a broad network encompassing the bilateral frontal cortex (Brodmann areas 9, 11, 45, and 46) was observed during the encoding, while an increased activity in the left orbitofrontal cortex (Brodmann area 11) was observed during the retrieval. Increased HbO concentration in the superior parietal cortices and decreased HbO concentration in the inferior parietal cortices were observed during encoding while dominant activation of left PFC was found during retrieval only. Higher task difficulty was associated with greater neural activity in the bilateral prefrontal cortex and higher task novelty was associated with greater activation in occipital regions. Conclusion: Combining the PAL paradigm with *f*NIRS provided the means to differentiate neural activity characterising encoding and retrieval. Therefore, the *f*NIRS may have the potential to complete EM assessments in clinical settings.

## 1. Introduction

Episodic memory (EM) refers to the process of currently retrieving events from the past [1,2]. EM incorporates two main phases: (a) an encoding stage, which contributes to the formation of a new memory trace, and (b) a retrieval stage, which refers to the conscious remembering of past events. EM is an essential process for various higher cortical functions such as judgment and decision making [1,2]. EM performance has been reported to be particularly sensitive to aging and pathological conditions such as amnesia, mild cognitive impairment (MCI), and Alzheimer’s disease (AD) [3,4]. Notably, a recent study indicated subjective memory complaints in 14% of younger adults (*N* = 4425) aged between 18 and 39 [5]. The progressive decline of EM has detrimental effects on multiple daily life outcomes including educational success and work performance [6,7]. Furthermore, because of the associated medical cost, EM impairment has been shown to increase financial pressures on families of affected people [8,9,10].

Over the last two decades, the paired-associate learning (PAL) paradigm has become one of the most widely used paradigms to assess EM performance [11,12,13]. PAL paradigm usually involves recollection of objects after learning a series of associations between stimuli (e.g., faces and names). Given that the difficulty and novelty of the paradigm can be easily manipulated by means of item–item associations, it can be adapted to the functional levels of targeted clinical populations. In addition, the paradigm does not include complex language requirements, and it has been widely used to examine EM performances in both healthy participants and individuals with apparent memory impairments [14,15].

Based on the results of previous functional magnetic resonance imaging (*f*MRI) researches and lesion studies, specific brain regions (i.e., prefrontal, parietal and occipital cortices, and temporal hippocampal areas) have been suggested to be engaged in the process of EM [16,17,18]. According to the transfer-appropriate processing (TAP) theory, retrieval success depends on the successful encoding. Specifically, the extent of encoding-related activation in regions engaged during both encoding and retrieval has been associated with subsequent memory success [19]. However, this reengagement phenomenon in EM has not been well proved by neuroimaging evidence. Moreover, it remains largely elusive whether the novelty (repetition times; repeated pairs vs. novel pairs) and difficult levels (number of characters in a name; two-word pairs vs. three-word pairs) of stimulus can influence the accuracy rate of retrieval. To elucidate the neural mechanisms underlying the retrieval successes, the use of functional near-infrared spectroscopy (*f*NIRS) has been recently suggested as it provides the means to identify brain functioning to investigate the central and autonomous nervous system in more ecological and clinically usable contexts [20].

Recently, the combination of behavioral assessment with *f*NIRS recordings has been broadly and efficiently applied in the exploration of cognitive performances and associated brain correlates [21]. Compared with electroencephalogram (EEG) and *f*MRI, *f*NIRS is less sensitive to movement artifacts and has fewer contraindications (e.g., allow the participation of individuals with metallic implants such as braces). Thus, *f*NIRS technology enables researchers to monitor the brain activity of children, healthy adults, as well as clinical population (e.g., MCI, AD) with less stress, higher ecological validity, and fewer costs, thus allowing a better integration into the clinical practice. Regarding the PAL paradigm, *f*NIRS allows investigators to record the verbal answers and EM-related cortical brain activity simultaneously [22]. Given its advantages, we aimed to combine the PAL paradigm with *f*NIRS to examine differences in oxygenated–hemoglobin concentrations associated with different aspects of paradigm (i.e., memory phases, novelty, and difficulty level of face-name pairs) among the male college students. Specifically, we hypothesized that the encoding and retrieval should be characterized by different brain responses among the frontal and parietal cortices in spite of partial activation overlap [23,24,25], allowing to differentiate the two phases of EM. As more cognitive resources and efforts are required when performing a novel task [26] and with increasing difficulty levels [27,28,29], we also hypothesized that greater activation would be observed among pairs of trials presenting contrasting novelty (vs. repeated/same pair) and difficulty levels.

## 2. Methods

### 2.1. Participants

Based on a priori sample size calculation (*α* = 0.05, effective size *d*_z_ = 0.8) [22], 22 male students from Shenzhen University (China; age: *M* = 20.55, *SD* = 1.62) participated in this study. Inclusion criteria were (a) normal visual, hearing, and physical function to complete the experiment; (b) right-handedness; (c) absence of mental health disorders or cognitive impairments; (d) no dependence on alcohol, nicotine, coffee, or drugs. Written informed consent forms were signed by all individuals before participation. This study was approved by Shenzhen University Institutional Review Board, with protocol (PN-2020-034) performed in accordance with the latest revision of the Declaration of Helsinki.

### 2.2. Experimental Procedure

**Figure 1:**
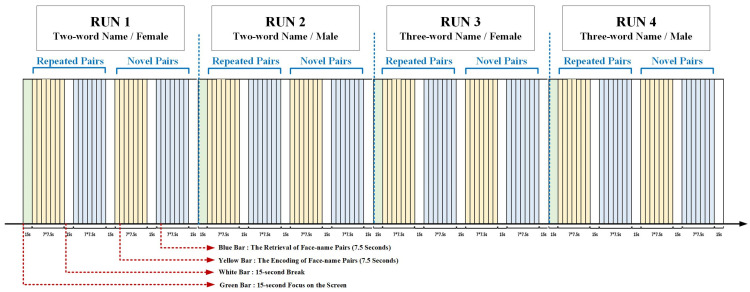
face-name PAL Paradigm.

We adopted the face-name PAL paradigm from a previous *f*MRI study (Figure 1) [30]. In the present study, the neutral faces were acquired from the CAS-PEAL Large Scale Chinese Face Database and were presented on the lab computer. There were 4 runs in the present paradigm. In the first two runs, two-word names were presented, with (a) run 1: female face-name pairs and (b) run 2: male face-name pairs. In the last two runs, three-word names were presented, with (a) run 3: female face-name pairs and (b) run 4: male face-name pairs. Each run started with a 15-s baseline, and consisted of 4 blocks presented following the order of encoding (repeated), retrieval (repeated), encoding (novel), and retrieval (novel) face-name pairs. In each block, 7 faces were randomly presented on the white background, each lasting for 4.5 s and with 3-s intervals between faces. Each block was followed by a 15-s reset period. In the novel trials, 7 different face-name pairs were randomly presented; in the repeated trials, 2 different face-name pairs were randomly represented (at least 3 times for each pair). The two-word Chinese names and the three-word Chinese names (higher difficult level) represented different difficulty levels in our study. In the encoding phase, the participants were asked to try their best to remember the face-name pairs. In the retrieval phase, the faces which appeared in the encoding phases could be presented, and the participants were required to report the names associated with the presented faces within 4.5 s. The accuracy (accuracy = the number of correct answers/the number of total face-name pairs * 100%) was noted by investigators.

Before the experiment, the entire procedure was explained in detail by the investigator and the participants were given specific instructions on how to remember the face-name matches for later testing. During the experiment, the participants were required to sit in front of the computer (45 cm) without extraneous movements for about 24 min and the *f*NIRS data were collected simultaneously. At the beginning, a white fixation cross (+) was presented in the center of the visual field on a black background to ensure the concentration of participants on paradigm stimuli. During the encoding runs, seven faces with names printed underneath were shown, and the participants were asked to memorize as accurately as possible the face-name pairs presented on the screen. In the retrieval runs, a face without a name appeared on the screen, and the participants were required to report the paired name within the given time (7.5 s). During the rest periods, a 15-s black screen was displayed. The participants’ responses were recorded for further analysis.

#### 2.2.1. *f*NIRS Data Acquisition

**Figure 2:**
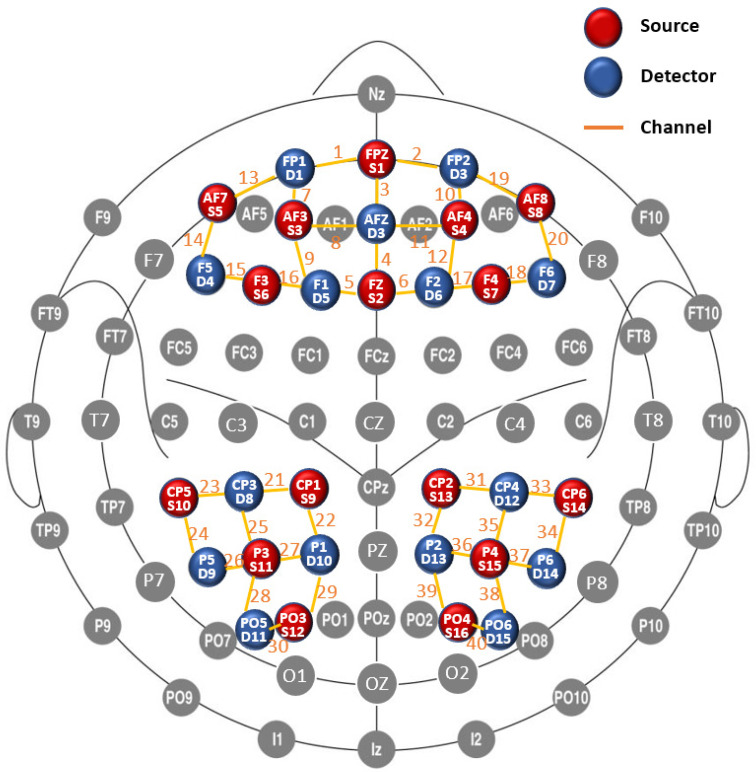
Location of sources, detectors and channels on the 2D Plane. Note 1. MNI coordinates. channel 01 (−13, 70, −12), channel 02 (14, 71, −12), channel 03 (0, 68, 8), channel 04 (1, 55, 40), channel 05 (−10, 44, 54), channel 06 (13, 44, 54), channel 07 (−27, 68, 0), channel 08 (−14, 70, 19), channel 09 (−23, 58, 35), channel 10 (28, 69, −1), channel 11 (16, 71, 19), channel 12 (27, 57, 33), channel 13 (−37, 61, −11), channel 14 (−50, 47, 3), channel 15 (−47, 39, 30), channel 16 (−30, 41, 46), channel 17 (33, 41, 47), channel 18 (48, 40, 31), channel 19 (39, 64, −10), channel 20 (51, 49, 5), channel 21 (−38, −32, 71), channel 22 (−22, −46, 76), channel 23 (−64, −36, 46), channel 24 (−64, −58, 22), channel 25 (−50, −53, 57), channel 26 (−51, −75, 33), channel 27 (−33, −66, 63), channel 28 (−40, −87, 30), channel 29 (−24, −80, 54), channel 30 (−28, −98, 19), channel 31 (42, −30, 70), channel 32 (29, −48, 74), channel 33 (65, −32, 49), channel 34 (65, −53, 31), channel 35 (53, −49, 57), channel 36 (38, −65, 61), channel 37 (53, −69, 40), channel 38 (44, −83, 32), channel 39 (31, −82, 48), channel 40 (33, −97, 14). Note 2. Location. prefrontal cortex: channel 1–20; superior parietal cortex: channel 22, 32; inferior parietal cortex: channel 21–27, 29, 31–37, 39; middle occipital cortex: channel 28, 30, 38, 40.

A 40-channel *f*NIRS system (Danyang Huichuang Medical Equipment Co. Ltd., China) was used to measure the relative change in oxygenated hemoglobin (HbO). Beta values were computed within the bilateral prefrontal, superior parietal, inferior parietal, and middle occipital cortices. The *f*NIRS system operated at two wavelengths (750 and 850 nm) and recorded changes at a sample rate of 7 Hz. In total, 16 sources and 15 detectors were used and the optodes were placed in the cap based on the international 10–5 system for EEG electrode placement (Figure 2), with a black overcap covering all the optodes to avoid interference from environmental light (Babak S et al., 2010). The uniform distance between neighboring optodes was 3 cm. The 3D positions (MNI standard coordinates) of the sources and detectors, as well as the reference optodes, were calculated via a 3D digitizer (PATRIOT, Polhemus), which is based on a four-point positioning algorithm in 3D space relying on received signal strength indication.

We mapped the *f*NIRS channels to the corresponding brain regions according to their MNI coordinates, as shown in Figure 2 (prefrontal cortex: channel 1–20; superior parietal cortex: channel 22, 32; inferior parietal cortex: channel 21–27, 29, 31–37, 39; middle occipital cortex: channel 28, 30, 38, 40). Additionally, the coordinates were checked using the *f*NIRS Optodes’ Location Decider (fOLD) [31].

#### 2.2.2. *f*NIRS Data Preprocessing

The raw *f*NIRS data were recorded during the face-name PAL paradigm using the NIRSmart acquisition software and then processed via the NIRSpark analyzing software. During the data preprocessing, the unrelated time intervals and artifacts induced by motion and environment were eliminated (automatic motion correction of NIRSpark analyzing software; the standard deviation of threshold = 6.0; the amplitude of threshold = 0.5). The light intensities were converted to optical densities and blood oxygen concentrations through the modified Beer–Lambert law. A bandpass filter (0.01–0.1 Hz) was then applied to remove both noise and interference signals (heart rate, breathing rate, and Mayer waves) [32]. The initial time of the hemodynamic response function (HRF) was set to −2 s (i.e., baseline state) and the end time to 50 s (i.e., task state for a single block). The oxygenated HRF was averaged for each channel across the four blocks.

### 2.3. Data Analysis

**Figure 3:**
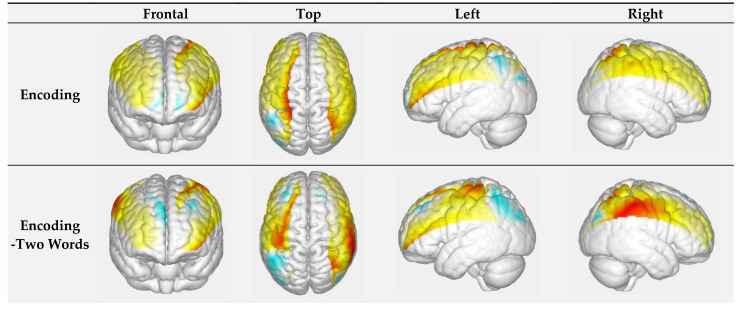

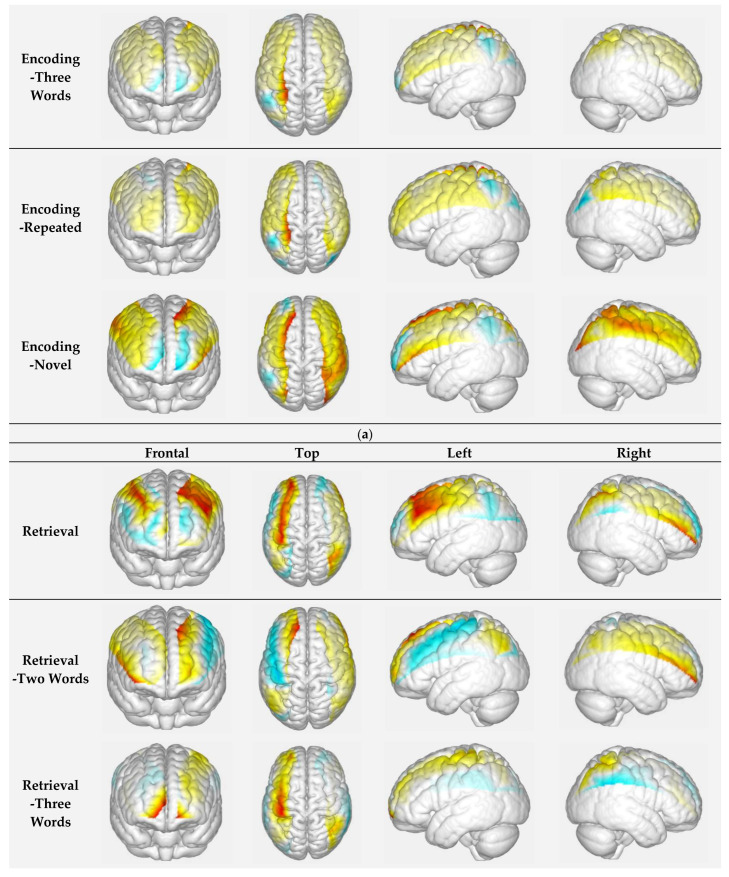

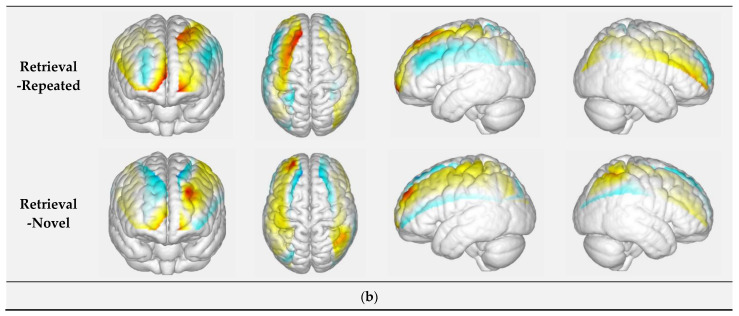
(**a**) Visualization of beta values in the encoding condition; (**b**) visualization of beta values in the retrieval. The warmer color represents a higher beta value (positive), whereas the cooler represents a lower beta value (negative).

The generalized linear model (GLM) was employed to analyze channel-wise hemodynamic responses induced by the face-name PAL paradigm. The GLM established a canonical HRF for each experiment condition and each participant, and calculate the degree of matching between the experimental and ideal HRF values. GLM models were used to analyze HbO concentration, with the stage (encoding/retrieval), novelty (novel/repeated), and difficult level of face-name pairs (two-word name/three-word name) as independent measures. The beta value represented how much a regressor (e.g., experimental manipulation) contributed to the *f*NIRS signal and whether the HRF model was reliable (Pinti P et al., 2017). Diffuse optical topography (Figure 3) of beta value was generated by EasyTopo [33,34], a toolbox running through MatLab software. Additionally, mean activation of hemodynamic state (HbO concentration) was also computed and exported for postprocessing analyses. In this study, only HbO signals calculated by the modified Beer–Lambert law were used. HbO is less influenced by physiological noise and better reflect changes in the regional cerebral blood oxygenation induced by stimuli when compared to deoxygenated hemoglobin (Hb) [35]. The path length factor was set at 6 for both 740 nm and 850 nm waves.

For the exported data (HbO concentration and beta value), the analysis was performed using IBM SPSS (v.23.0). The normality distribution of data was checked by the Shapiro–Wilk test, as recommended for small sample sizes [36,37]. Then, the paired-sample *t*-tests (e.g., encoding vs. retrieval, repeated pairs vs. novel pairs (novelty), two-word pairs vs. three-word pairs (difficulty level)) were used for data of normal distribution, and the Wilcoxon signed-rank tests were used for the data of non-normal distribution, with a significance level set at α = 0.05. As recommended for *f*NIRS studies, the false discovery rate (FDR) was used to account for the multiple comparison problem (i.e., type I error accumulation) [38,39]. For the behavioral data, Pearson’s correlation was used to test the associations between the accuracy of cognitive paradigm (number of correct answers) and beta value [40]. We rated the correlation coefficients as follows: 0 to 0.19: no correlation; 0.2 to 0.39: low correlation, 0.40 to 0.59: moderate correlation; 0.60 to 0.79: moderately high correlation; ≥0.80: high correlation.

## 3. Results

Table 1 shows the significances of HbO concentration among channels in the different task conditions. The channels with significant differences in beta values (baseline vs. task condition) are shown in Table 2. Finally, the visualization of beta values in the encoding and retrieval phases is illustrated in Figure 3.

### 3.1. Encoding and Retrieval Phases

During the encoding, HbO concentration showed significant increases from baseline in the left superior parietal cortex (channel 22; FDR-corrected *p*-value = 0.038); and the significant decreases were observed in bilateral prefrontal cortices and left inferior parietal cortex (channel 1, 3, 17, 18, 20, 25, 26; FDR-corrected *p*-values = 0.048, 0.048, 0.012, 0.048, 0.029, 0.000, 0.012). During the retrieval phase, a significant increase from baseline of HbO concentration was only observed in the left prefrontal cortex (channel 1; FDR-corrected *p*-value = 0.048).

In the phase of encoding, the highest beta value was 0.611 located in the left superior parietal cortex, and the lowest beta value was reported in the middle occipital cortex (left: −0.147; right: 0.020). For the retrieval phase, the highest beta value (0.231) was found in the left prefrontal cortex while the lowest one (−0.183) in the left superior parietal cortex. Compared with retrieval, significantly higher beta values during encoding were observed in the right prefrontal cortex (channel 10,12; FDR-corrected *p*-values = 0.022, 0.022), while significantly lower beta values were observed in the left prefrontal cortex (channel 1; FDR-corrected *p*-value = 0.021) and right middle occipital cortex (FDR-corrected *p*-value = 0.021).

#### 3.1.1. Encoding of Faces with Different Difficulty-Level Names: Two-Word Names vs. Three-Word Names

During the encoding of faces with two-word names, the significant decreases of HbO concentration were observed in bilateral prefrontal cortices, left inferior parietal cortex, and left middle occipital cortex (channel 15, 17, 25, 26, 30; FDR-corrected *p*-values = 0.026, 0.034, 0.010, 0.012, 0.048). For the encoding of faces with three-word names, significant differences of HbO concentration were shown in the right prefrontal cortex and bilateral inferior parietal cortices (increase from baseline: channel 33; FDR-corrected *p*-value = 0.035) (decreases from baseline: channel 19, 20, 23, 37; FDR-corrected *p*-values = 0.035, 0.035, 0.010, 0.048). Compared with the encoding of faces with two-word names, the encoding of faces with three-word names showed a significant increase of beta value in the bilateral prefrontal cortex (channel 4; FDR-corrected *p*-values = 0.032).

#### 3.1.2. Encoding of Face at Different Novelty Levels: Repeated Faces vs. Novel Faces

During the encoding of repeated faces, HbO concentration showed a significant increase in the left superior parietal cortex (Channel 22; FDR-corrected *p*-value = 0.035) and decreases in right prefrontal cortex and left inferior parietal cortex (Channel 18, 25; FDR-corrected *p*-values = 0.026, 0.010). For the encoding of novel faces, significant decreases were observed in the right prefrontal cortex and left inferior parietal cortex (Channel 2,17, 23, 26; FDR-corrected *p*-values = 0.048, 0.012, 0.026, 0.035). Compared with the encoding with repeated faces, significant differences of beta values were observed in the right prefrontal cortex (lower beta value in channel 11; FDR-corrected *p*-value = 0.022) and right middle occipital cortex (higher beta value in channel 38; FDR-corrected *p*-value = 0.034) during the encoding with novel faces.

#### 3.1.3. Retrieval of Faces with Different Difficulty-Level Names: Two-Word Names vs. Three-Word Names

During the retrieval of faces with two-word names, there was no significant decrease or increase from baseline in HbO concentration. During the retrieval of faces with three-word names, HbO showed significant increases in the Brodmann area 11 of bilateral prefrontal cortices (channel 7, 19; FDR-corrected *p-*values = 0.031, 0.035) and decrease in the Brodmann area 46 of the right prefrontal cortex (channel 20; FDR-corrected *p*-value = 0.031). Between these two task conditions, no significant difference of beta value was observed in any brain regions.

#### 3.1.4. Retrieval of Face at Different Novelty Levels: Repeated Faces vs. Novel Faces

During the retrieval of repeated and novel faces, no channel showed a significant increase or decrease of HbO concentration in any brain region. Compared with the retrieval with repeated faces, significant differences of beta values were observed in the left prefrontal cortex (channel 5; FDR-corrected *p*-value = 0.021), left middle occipital cortex (channel 30; FDR-corrected *p*-value = 0.036), right inferior parietal cortex (channel 31; FDR-corrected *p*-value = 0.046) and right superior parietal cortex (channel 32; FDR-corrected *p*-value = 0.022) during the retrieval of novel faces.

### 3.2. Correlations between Memory Performance and Brain Activation (Beta Value)

No correlation was found between the accuracy of retrieval and brain activation during encoding (Table S1). During the retrieval of novel faces, negative correlations were observed between the accuracy of retrieval and beta values (right superior parietal cortex (channel 32): FDR-corrected *p*-values = 0.020; r = −0.573).

## 4. Discussion

The present study used *f*NIRS to examine the brain mechanisms underlying memory encoding and retrieval in a sample of male college students. Compared to baseline, the *f*NIRS results showed differential changes in brain activation in the PFC between the encoding and retrieval processes of episodic memory (EM). In particular, the encoding process was characterized by decreases in HbO, suggesting deactivations in broader subregions of the bilateral PFC (Brodmann area 9, 11, 45, 46). In contrast, the retrieval process was characterized by increases in HbO in the left orbitofrontal cortex (Brodmann area 11). Compared with retrieval, significantly higher beta values during encoding were observed in the right prefrontal cortex while significantly lower beta values were observed in the left prefrontal cortex and right middle occipital cortex. These outcomes were partially consistent with previous *f*MRI [16,17,18] and *f*NIRS [22] studies. Overall, our findings indicate the *f*NIRS can be a useful tool to model brain activation patterns during memory-related tasks.

Previous *f*MRI studies reported increased activation of PFC during encoding [41,42,43]. However, a recent *f*NIRS study [22] reported decreased prefrontal activity during encoding, which was consistent with our outcomes. The differences between *f*MRI and *f*NIRS can be explained by the fact that *f*MRI measures the deeper brain regions, including those in the prefrontal cortex. Regarding the dominant role of left PFC in the retrieval, at least two factors can explain this phenomenon. First, the activation of the left PFC–precuneus network resulted from item–item associations [44]. Secondly, the contribution of left PFC to acquire semantic knowledge was used in the face-name PAL paradigm [45,46,47].

The HbO status during encoding was opposite in the superior (increased activation) and inferior (Brodmann area 39, 40; decreased activation) parietal cortices. Notably, the activated superior parietal cortex is an essential component of the dorsal attention network (DAM) and contributes to the top-down modulation of sensory [48]. Likewise, the deactivated angular gyrus (i.e., Brodmann area 39), which is part of the default mode network (DMN), has been reported to be involved mainly in passive states [48,49,50]. Based on these past findings, it could be argued that memory encoding may require higher attentional processes and thereby may inhibit the function of DMN. Importantly, accumulating evidence pinpoint the important role of parietal DMN nodes in EM retrieval. Specifically, it has been suggested that the task-evoked activity first induces the close coupling of intra-DMN, para-hippocampal, and medial temporal regions and then activates the extra-DMN nodes to make a final decision [51,52]. Therefore, it can be drawn that the parietal cortex differentially responds to memory phases and is in coordination with intra/extra-DMN areas for different purposes.

In this study, regarding faces with different levels of naming difficulty, compared with the blocks with two-word names, the ones with three-word names generated greater beta values and HbO concentration levels in the bilateral prefrontal cortices (Brodmann 9, 10, 11) during both the encoding and retrieval processes. Additionally, during retrieval, the left hemisphere activation was observed to be more dominant among pairs with higher difficulty levels. These results are in line with previous neuroimaging studies [51,52] and fit well within the “cortical asymmetry of reflective activity (CARA)” hypothesis [53], demonstrating that the left prefrontal cortex is more likely to engage in the more complex and reflectively demanding test. The complexity and reflective demand of the task include detailed and deliberative evaluation, analysis, and maintenance of the input information as well as the self-cueing initiation for additional information. Furthermore, in the more difficult level of the task, the selection of relevant information and the inhibition of irrelevant information also requires the participation of the left prefrontal cortex [51,54,55,56].

A successful EM retrieval relies on the rapid reactivation of sensory (i.e., visual) information presented in the encoding process, a process that relies on the occipital cortex [57], which plays a key role in visual processing [57]. In the present study, the repeated pairs generated lower activation in the occipital cortex, compared to the novel pairs. This result is in agreement with the findings of a previous *f*MRI study observing that the repetition of items suppressed the occipital visual cortex activity [58,59]. It is reported that such repetition suppression arises from stimulus-specific expectations. In particular, the visual cortex (i.e., visual area 1) is likely to produce a weaker response for the expected stimulus relative to unexpected things [60].

According to the transfer-appropriate processing (TAP) theory, people tend to deal with a task faster and more efficiently if the associated stimulus has been experienced before. In terms of memory, this phenomenon can be explained as follows: the success of retrieval depends on the successful encoding processing [61,62,63]. It is hypothesized that key brain areas during encoding can reengage the retrieval process [64]. Our results partially matched with the TAP hypothesis. During the encoding and retrieval of three-word names, Brodmann area 46 was deactivated in a systematic fashion. Likewise, we found that participants did better on the repeated trials only in cases where the degree of activation (or deactivation) in several regions was similar in the encoding and retrieval phases.

### Limitations

Due to the limitation of *f*NIRS penetration depth, the brain activities from the temporal hippocampal area were not available for observation, as noted in previous *f*MRI studies. Due to the limitation in optode number, we could not assess the activation of the whole occipital lobe. However, the primary visual cortex (visual area 1; Brodmann area 17) and partial extrastriate areas (visual area 2–3; Brodmann area 18) were well covered by the channel setup in this study, which enables us to explore the basic role of the visual system in EM. Additionally, we did not observe the correlation between accuracy of retrieval and brain activation during encoding in the present study. In addition, future studies using *f*NIRS to investigate EM-related brain activation patterns should use data processing techniques to remove artifacts arising from systemic physiological changes (e.g., short-separation channel regression to account for changes in superficial blood flow) in order to reduce the likelihood of false-positive findings [65,66]. Moreover, considering the limitation of small sample size, as well as a single-sex and narrow-age group (male university students) in this study, a larger sample and wider-scale population will be required to test the present outcomes in the further studies.

## 5. Conclusions

In conclusion, the results of the present study demonstrated that the encoding and retrieval of EM are associated with distinct patterns of prefrontal, parietal, and middle occipital activation/deactivation. In addition, the paradigm difficulty and the novelty play modulating roles in the engagement of brain substrates. The opposite brain responses of superior and inferior parietal cortices during encoding and the dominant activation of left PFC during retrieval allow us to differentiate the two phases of EM using *f*NIRS technology during a face-name PAL paradigm. Moreover, these cerebral correlates findings were reinforced by the observation of significant correlations between brain activation patterns and behavioral performance (i.e., accuracy) during the retrieval process that was reinforced. These findings suggest that *f*NIRS may represent a valuable tool to investigate the brain processes engaged in EM.

## Figures and Tables

**Table 1:** The significance of HbO concentration in different conditions (i.e., baseline vs. task).

Conditions	Channel	Brodmann Area	Location	Baseline (Rest)		Task		FDR Corrected *p*-Value
				Mean	SD	Mean	SD	
Encoding	C1	11	Left Prefrontal Cortex	−0.001	0.011	−0.052	0.337	0.048
	C3	/	Prefrontal Cortex	−0.002	0.012	−0.050	0.314	0.048
	C17	9	Right Prefrontal Cortex	−0.001	0.016	−0.102	0.451	0.012
	C18	45		0.000	0.009	−0.069	0.455	0.048
	C20	46		0.000	0.008	−0.049	0.258	0.029
	C22	/	Left Superior Parietal Cortex	0.003	0.028	0.345	2.070	0.038
	C25	40	Left Inferior Parietal Cortex	−0.002	0.014	−0.219	0.815	0.000
	C26	39		−0.002	0.013	−0.198	0.868	0.012
Encoding -Two Words	C15	45	Left Prefrontal Cortex	0.000	0.008	−0.135	0.468	0.026
	C17	9	Right Prefrontal Cortex	0.000	0.009	−0.104	0.425	0.034
	C25	40	Left Inferior Parietal Cortex	0.000	0.012	−0.268	0.748	0.010
	C26	39		−0.004	0.012	−0.305	0.895	0.012
	C30	18	Left Middle Occipital Cortex	−0.002	0.012	−0.184	0.842	0.048
Encoding -Three Words	C19	11	Right Prefrontal Cortex	0.000	0.005	−0.012	0.298	0.035
	C20	46		0.000	0.004	−0.042	0.283	0.035
	C23	40	Left Inferior Parietal Cortex	0.001	0.018	−0.045	1.745	0.010
	C33	40	Right Inferior Parietal Cortex	0.002	0.022	0.222	2.251	0.035
	C37	39		0.000	0.016	−0.023	1.460	0.048
Encoding -Repeated	C18	45	Right Prefrontal Cortex	0.000	0.008	−0.106	0.383	0.026
	C22	/	Left Superior Parietal Cortex	0.006	0.028	0.558	2.224	0.035
	C25	40	Left Inferior Parietal Cortex	0.000	0.013	−0.252	0.730	0.010
Encoding -Novel	C2	11	Right Prefrontal Cortex	−0.002	0.011	−0.085	0.383	0.048
	C17	9		−0.003	0.020	−0.135	0.394	0.012
	C23	40	Left Inferior Parietal Cortex	−0.004	0.018	−0.199	0.692	0.026
	C26	39		−0.004	0.015	−0.233	0.930	0.035
Retrieval	C1	11	Left Prefrontal Cortex	0.002	0.018	0.237	1.551	0.048
Retrieval -Two words	/		/	/	/	/	/	/
Retrieval -Three words	C7	11	Left Prefrontal Cortex	0.001	0.012	0.121	0.456	0.031
	C19	11	Right Prefrontal Cortex	0.000	0.006	0.091	0.374	0.035
	C20	46		−0.001	0.005	0.084	0.320	0.031
Retrieval -Repeated	/		/	/	/	/	/	/
Retrieval -Novel	/		/	/	/	/	/	/

Prefrontal cortex: channel 1–20; superior parietal cortex: channel 22, 32; inferior parietal cortex: channel 21–27, 29, 31–37, 39; middle occipital cortex: channel 28, 30, 38, 40. FDR = false discovery rate.

**Table 2:** The significance of beta values in different conditions.

**(1) Encoding vs. Retrieval**						
**Channel**	**Location**	**Encoding**		**Retrieval**		**FDR Corrected *p*-Value**
		**Mean**	**SD**	**Mean**	**SD**	
C1	Left Prefrontal Cortex	−0.179	0.926	0.881	1.936	0.021
C10	Right Prefrontal Cortex	0.217	0.291	−0.206	0.723	0.022
C12		0.181	0.283	−0.199	0.549	0.022
C40	Right Middle Occipital Cortex	0.066	0.843	0.109	0.818	0.021
**(2) Encoding (Two Words) vs. Encoding (Three Words)**						
**Channel**	**Location**	**Encoding** **(Two Words)**		**Encoding** **(Three Words)**		**FDR Corrected *p*-Value**
		**Mean**	**SD**	**Mean**	**SD**	
C4	Prefrontal Cortex	−0.390	1.174	0.456	1.002	0.032
**(3) Encoding (Repeated) vs. Encoding (Novel)**						
**Channel**	**Location**	**Encoding** **(Repeated)**		**Encoding** **(Novel)**		**FDR Corrected *p*-Value**
		**Mean**	**SD**	**Mean**	**SD**	
C11	Right Prefrontal Cortex	0.223	0.476	−0.002	0.274	0.022
C38	Right Middle Occipital Cortex	−0.713	1.530	0.342	1.199	0.034
**(4) Retrieval (Two Words) vs. Retrieval (Three Words)**						
**Channel**	**Location**	**Retrieval** **(Two Words)**		**Retrieval** **(Three Words)**		**FDR Corrected *p*-Value**
		**Mean**	**SD**	**Mean**	**SD**	
/	/	/	/	/	/	/
**(5) Retrieval (Repeated) vs. Retrieval (Novel)**						
**Channel**	**Location**	**Retrieval** **(Repeated)**		**Retrieval** **(Novel)**		**FDR Corrected *p*-Value**
		**Mean**	**SD**	**Mean**	**SD**	
C5	Left Prefrontal Cortex	0.956	2.210	−0.618	2.242	0.021
C30	Left Middle Occipital Cortex	−0.273	1.005	0.289	0.826	0.036
C31	Right Inferior Parietal Cortex	−0.052	0.344	0.178	0.310	0.046
C32	Right Superior Parietal Cortex	−0.150	1.183	0.120	1.015	0.022

FDR = false discovery rate.

## Data Availability

Data of this study were collected at Shenzhen University.

## Supplementary Materials

The following are available online at https://www.mdpi.com/article/10.3390/brainsci11070951/s1, Table S1: Accuracy in Retrieval Phase.

## References

[1] Schacter D.L., Gilbert D.T., Wegner D.M. (2009). Semantic and Episodic Memory, Psychology.

[2] Tulving E. (1983). Elements of Episodic Memory.

[3] Anderson K.E., Lynch K., Zarahn E., Scarmeas N., Van Heertum R., Sackeim H., Moeller J.R., Stern Y. (2005). H215O pet study of impairment of nonverbal recognition with normal aging. J. Neuropsychiatry Clin. Neurosci..

[4] Tromp D., Dufour A., Lithfous S., Pebayle T., Després O. (2015). Episodic memory in normal aging and alzheimer disease: Insights from imaging and behavioral studies. Ageing Res. Rev..

[5] Chen S.T., Siddarth P., Ercoli L.M., Merrill D., Torres-Gil F., Small G.W. (2014). Modifiable risk factors for alzheimer disease and subjective memory impairment across age groups. PLoS ONE.

[6] Conway M.A., Gardiner J.M., Perfect T.J., Anderson S.J., Cohen G.M. (1997). Changes in memory awareness during learning: The acquisition of knowledge by psychology undergraduates. J. Exp. Psychol. Gen..

[7] Herbert D.B.M., Burt J.S. (2004). What do students remember? Episodic memory and the development of schematization. Appl. Cogn. Psychol..

[8] Irish M., Lawlor B.A., Coen R.F., O’Mara S.M. (2011). Everyday episodic memory in amnestic mild cognitive impairment: A preliminary investigation. BMC Neurosci..

[9] Paradise M., McCade D., Hickie I.B., Diamond K., Lewis S., Naismith S. (2015). Caregiver burden in mild cognitive impairment. Aging Ment. Health.

[10] Wong W. (2020). Economic burden of alzheimer disease and managed care considerations. Am. J. Manag. Care..

[11] Blackwell A.D., Sahakian B., Vesey R., Semple J.M., Robbins T.W., Hodges J.R. (2004). Detecting dementia: Novel neuropsychological markers of preclinical alzheimer’s disease. Dement. Geriatr. Cogn. Disord..

[12] De Jager C., Blackwell A.D., Budge M.M., Sahakian B.J. (2005). Predicting cognitive decline in healthy older adults. Am. J. Geriatr. Psychiatry.

[13] Fowler K.S., Saling M.M., Conway E.L., Semple J.M., Louisp W.J. (2002). Paired associate performance in the early detection of dat. J. Int. Neuropsychol. Soc..

[14] Crystal J.D. (2009). Elements of episodic-like memory in animal models. Behav. Process..

[15] Eichenbaum H., Alvarez P., Ramus S.J. (2000). Animal Models of Amnesia.

[16] Glahn D.C., Robinson J.L., Tordesillas-Gutierrez D., Monkul E.S., Holmes M.K., Green M., Bearden C. (2010). Fronto-temporal dysregulation in asymptomatic bipolar i patients: A paired associate functional mri study. Hum. Brain Mapp..

[17] Köhler S., Paus T., Buckner R.L., Milner B. (2004). Effects of left inferior prefrontal stimulation on episodic memory formation: A two-stage fmri-rtms study. J. Cogn. Neurosci..

[18] Sidhu M.K., Stretton J., Winston G., Bonelli S., Centeno M., Vollmar C., Symms M., Thompson P.J., Koepp M.J., Duncan J.S. (2013). A functional magnetic resonance imaging study mapping the episodic memory encoding network in temporal lobe epilepsy. Brain.

[19] Rugg M.D., Johnson J.D., Uncapher M.R. (2015). Encoding and Retrieval in Episodic Memory: Insights from Fmri.

[20] Adorni R., Gatti A., Brugnera A., Sakatani K., Compare A. (2016). Could fNIRS Promote Neuroscience Approach in Clinical Psychology?. Front. Psychol..

[21] Yücel M.A., Lühmann A.V., Scholkmann F., Gervain J., Dan I., Ayaz H., Boas D., Cooper R.J., Culver J., Elwell C.E. (2021). Best practices for fnirs publications. Neurophotonics.

[22] Jahani S., Fantana A.L., Harper D., Ellison J.M., Boas D.A., Forester B.P., Yücel M.A. (2017). Fnirs can robustly measure brain activity during memory encoding and retrieval in healthy subjects. Sci. Rep..

[23] Cabeza R., Nyberg L. (2000). Imaging cognition ii: An empirical review of 275 pet and fmri studies. J. Cogn. Neurosci..

[24] Gui P., Ku Y., Li L., Li X., Bodner M., Lenz F.A., Wang L., Zhou Y.-D. (2017). Neural correlates of visuo-tactile crossmodal paired-associate learning and memory in humans. Neuroscience.

[25] Preston A.R., Eichenbaum H. (2013). Interplay of hippocampus and prefrontal cortex in memory. Curr. Biol..

[26] Hunkin N.M., Mayes A.R., Gregory L.J., Nicholas A.K., Nunn J.A., Brammer M.J., Bullmore E.T., Williams S.C. (2002). Novelty-related activation within the medial temporal lobes. Neuropsychologia.

[27] Nyberg L., Tulving E., Habib R., Nilsson L.G., Kapur S., Houle S., Cabeza R., McIntosh A.R. (1995). Functional brain maps of retrieval mode and recovery of episodic information. Neuroreport.

[28] Schacter D.L., Reiman E., Uecker A., Roister M.R., Yun L.S., Cooper L.A. (1995). Brain regions associated with retrieval of structurally coherent visual information. Nature.

[29] Tulving E., Markowitsch H.J., Craik F.I.M., Habib R., Houle S. (1996). Novelty and familiarity activations in pet studies of memory encoding and retrieval. Cereb. Cortex..

[30] Sperling R.A., Bates J.F., Cocchiarella A.J., Schacter D.L., Rosen B.R., Albert M.S. (2001). Encoding novel face-name associations: A functional mri study. Hum. Brain Mapp..

[31] Zimeo Morais G.A., Balardin J.B., Sato J.R. (2018). fNIRS Optodes’ Location Decider (fOLD): A toolbox for probe arrangement guided by brain regions-of-interest. Sci. Rep..

[32] Pinti P., Scholkmann F., Hamilton A., Burgess P., Tachtsidis I. (2018). Current status and issues regarding pre-processing of fnirs neuroimaging data: An investigation of diverse signal filtering methods within a general linear model framework. Front. Hum. Neurosci..

[33] Tian F., Kozel F.A., Yennu A.S., Croarkin P.E., McClintock S.M., Mapes K.S., Husain M.M., Liu H. (2012). Test-retest assessment of cortical activation induced by repetitive transcranial magnetic stimulation with brain atlas-guided optical topography. J. Biomed. Opt..

[34] Tian F., Lin Z.J., Liu H. Easytopo: A toolbox for rapid diffuse optical topography based on a standard template of brain atlas. Proceedings of the SPIE. 8578: 85782J.

[35] Strangman G., Culver J.P., Thompson J.H., Boas D.A. (2002). A quantitative comparison of simultaneous bold fmri and nirs recordings during functional brain activation. Neuroimage.

[36] Ghasemi A., Zahediasl S. (2012). Normality tests for statistical analysis: A guide for non-statisticians. Int. J. Endocrinol. Metab..

[37] Shapiro S.S., Wilk M.B., Chen H.J. (1968). A comparative study of various tests for normality. J. Am. Stat. Assoc..

[38] Benjamini Y., Hochberg Y. (1995). Controlling the false discovery rate: A practical and powerful approach to multiple testing. J. R. Stat. Soc. Ser. B (Methodol.).

[39] Singh A.K., Dan I. (2006). Exploring the false discovery rate in multichannel nirs. Neuroimage.

[40] Zhu W. (2016). P < 0.05, < 0.01, < 0.001, < 0.0001, < 0.00001, < 0.000001, or < 0.0000001. J. Sport Health Sci..

[41] Blumenfeld R.S., Ranganath C. (2007). Prefrontal cortex and long-term memory encoding: An integrative review of findings from neuropsychology and neuroimaging. Neuroscientist.

[42] Demb J.B., Desmond J.E., Wagner A.D., Vaidya C.J., Glover G.H., Gabrieli J.D. (1995). Semantic encoding and retrieval in the left inferior prefrontal cortex: A functional mri study of task difficulty and process specificity. J. Neurosci..

[43] Opitz B., Mecklinger A., Friederici A.D. (2000). Functional asymmetry of human prefrontal cortex: Encoding and retrieval of verbally and nonverbally coded information. Learn. Mem..

[44] Lundstrom B.N., Petersson K.M., Andersson J., Johansson M., Fransson P., Ingvar M. (2003). Isolating the retrieval of imagined pictures during episodic memory: Activation of the left precuneus and left prefrontal cortex. Neuroimage.

[45] Friederici A.D. (2002). Towards a neural basis of auditory sentence processing. Trends Cogn. Sci..

[46] Hagoort P., Hald P., Petersson K.M. (2002). Semantic vs. world knowledge integration during sentence comprehension. J. Cogn. Neurosci. Suppl..

[47] Wagner A.D., Paré-Blagoev E.J., Clark J., Poldrack R.A. (2001). Recovering meaning: Left prefrontal cortex guides controlled semantic retrieval. Neuron.

[48] Sestieri C., Shulman G.L., Corbetta M. (2017). The contribution of the human posterior parietal cortex to episodic memory. Nat. Rev. Neurosci..

[49] Buckner R.L., Andrews-Hanna J.R., Schacter D.L. (2008). The brain’s default network: Anatomy, function, and relevance to disease. Ann. N. Y. Acad. Sci..

[50] White T.P., Jansen M., Doege K., Mullinger K., Park S.B., Liddle E.B., Gowland P., Francis S.T., Bowtell R., Liddle P.F. (2013). Theta power during encoding predicts subsequent-memory performance and default mode network deactivation. Hum. Brain Mapp..

[51] Sestieri C., Corbetta M., Romani G.L., Shulman G.L. (2011). Episodic memory retrieval, parietal cortex, and the default mode network: Functional and topographic analyses. J. Neurosci..

[52] Wagner A.D., Shannon B.J., Kahn I., Buckner R.L. (2005). Parietal lobe contributions to episodic memory retrieval. Trends Cogn. Sci..

[53] Nolde S.F., Johnson M.K., Raye C.L. (1998). The role of prefrontal cortex during tests of episodic memory. Trends Cogn. Sci..

[54] Fletcher P.C., Henson R.N. (2001). Frontal lobes and human memory: Insights from functional neuroimaging. Brain.

[55] Pinti P., Merla A., Aichelburg C., Lind F., Power S., Swingler E., Hamilton A., Gilbert S., Burgess P.W., Tachtsidis I. (2017). A novel glm-based method for the automatic identification of functional events (aide) in fnirs data recorded in naturalistic environments. Neuroimage.

[56] Thompson-Schill S.L., D’Esposito M., Kan I.P. (1999). Effects of repetition and competition on activity in left prefrontal cortex during word generation. Neuron.

[57] Huff T., Mahabadi N., Tadi P. (2020). Neuroanatomy, Visual Cortex.

[58] Qiao F., Zheng L., Li L., Zhu L., Wang Q. (2014). Reduced repetition suppression in the occipital visual cortex during repeated negative chinese personality-trait word processing. Scand. J. Psychol..

[59] Grill-Spector K., Henson R., Martin A. (2006). Repetition and the brain: Neural models of stimulus-specific effects. Trends Cogn. Sci..

[60] Utzerath C., John-Saaltink E.S., Buitelaar J., De Lange F.P. (2017). Repetition suppression to objects is modulated by stimulus-specific expectations. Sci. Rep..

[61] Franks J.J., Bilbrey C.W., Lien K.G., McNamara T.P. (2000). Transfer-appropriate processing (tap) and repetition priming. Mem. Cognit..

[62] Morris C.D., Bransford J.D., Franks J.J. (1977). Levels of processing versus transfer appropriate processing. J. Verbal Learn. Verbal Behav..

[63] Tulving E., Thomson D.M. (1973). Encoding specificity and retrieval processes in episodic memory. Psychol. Rev..

[64] Morcom A.M. (2014). Re-engaging with the past: Recapitulation of encoding operations during episodic retrieval. Front. Hum. Neurosci..

[65] Herold F., Wiegel P., Scholkmann F., Müller N.G. (2018). Applications of functional near-infrared spectroscopy (fnirs) neuroimaging in exercise-cognition science: A systematic, methodology- focused review. J. Clin. Med..

[66] Tachtsidis I., Scholkmann F. (2016). False positives and false negatives in functional near-infrared spectroscopy: Issues, challenges, and the way forward. Neurophotonics.

