# Supplementary material for: Episodic Memory Encoding and Retrieval in Face-Name Paired Paradigm: An fNIRS Study
Source: Brain Sci. 2021 Jul 19;11(7):951. doi: 10.3390/brainsci11070951 (PMC8305286; doi:10.3390/brainsci11070951)
Supplement: Supplementary file 1 [file brainsci-11-00951-s001.zip › brainsci-1230647-supplementary.pdf]

Supplementary Materials

Accuracy in Retrieval Phases

	Accuracy				
**Subject**	**Retrieval**	**Retrieval_twowords**	**Retrieval_threewords**	**Retrieval_same**	**Retrieval_novel**
**1**	55.36%	53.57%	57.14%	100.00%	10.71%
**2**	51.79%	53.57%	50.00%	100.00%	3.57%
**3**	57.14%	57.14%	57.14%	100.00%	14.29%
**4**	53.57%	50.00%	57.14%	100.00%	7.14%
**5**	53.57%	50.00%	57.14%	100.00%	7.14%
**6**	39.29%	25.00%	53.57%	75.00%	3.57%
**7**	69.64%	71.43%	67.86%	100.00%	39.29%
**8**	57.14%	60.71%	53.57%	100.00%	14.29%
**9**	53.57%	50.00%	57.14%	100.00%	7.14%
**10**	41.07%	32.14%	50.00%	75.00%	7.14%
**11**	58.93%	57.14%	60.71%	100.00%	17.86%
**12**	23.21%	46.43%	0.00%	42.86%	3.57%
**13**	62.50%	60.71%	64.29%	100.00%	25.00%
**14**	53.57%	53.57%	53.57%	100.00%	7.14%
**15**	44.64%	28.57%	60.71%	71.43%	17.86%
**16**	39.29%	28.57%	50.00%	75.00%	3.57%
**17**	62.50%	57.14%	67.86%	100.00%	25.00%
**18**	55.36%	53.57%	57.14%	96.43%	14.29%
**19**	55.36%	53.57%	57.14%	100.00%	10.71%
**20**	62.50%	57.14%	67.86%	89.29%	32.14%
**21**	58.93%	53.57%	64.29%	96.43%	21.43%
**22**	53.57%	53.57%	53.57%	96.43%	10.71%
